# Endometriosis and Infertility: Gynecological Examination Practical Guide

**DOI:** 10.3390/jcm14061904

**Published:** 2025-03-12

**Authors:** Alice Moïse, Milana Dzeitova, Laurent de Landsheere, Michelle Nisolle, Géraldine Brichant

**Affiliations:** 1 Department of Obstetrics and Gynecology, Hopital de La Citadelle, University of Liège, 4000 Liège, Belgium; ldelandsheere@chuliege.be (L.d.L.); michelle.nisolle@chuliege.be (M.N.); gbrichant@chuliege.be (G.B.); 2 University of Liège, 4000 Liège, Belgium; milana.dzeitova@student.uliege.be

**Keywords:** endometriosis, infertility, examination

## Abstract

Endometriosis, a prevalent gynecological condition affecting 10–15% of reproductive-age women, involves the growth of endometrial-like tissue outside the uterine cavity. This chronic inflammatory disease can significantly impact fertility by disrupting ovulation, tubal transport, and implantation. Clinical manifestations vary widely, ranging from asymptomatic cases to severe pelvic pain, dysmenorrhea, and dyspareunia. Accurate diagnosis remains challenging, often requiring a combination of patient history, clinical examination, and imaging studies. This paper will discuss the clinical approach to endometriosis during a first-line gynecological appointment, focusing on patient history, including detailed assessment of menstrual, pelvic, and bowel symptoms, and clinical examination; thorough gynecological examination, including abdominal and pelvic palpation, speculum examination, and bimanual examination; imaging evaluation (particularly of the role of ultrasound in identifying and characterizing endometriotic lesions, including the use of the #ENZIAN classification for deep infiltrating endometriosis and evaluation of fertility impact); and discussion of the Endometriosis Fertility Index (EFI) as a tool for assessing fertility potential. This comprehensive approach aims to guide clinicians in identifying and managing endometriosis effectively, improving patient outcomes and optimizing fertility management strategies. **Methods**: A literature search for suitable articles published from January 1974 to 2024 in the English language was performed using PubMed. **Results:** Endometriosis is associated with infertility rates ranging from 20% to 68%, with mechanisms including pelvic adhesions, chronic inflammation, and immune dysregulation. The revised American Society for Reproductive Medicine (rASRM) classification and #ENZIAN classification were identified as essential tools for staging and characterizing the disease. Transvaginal ultrasound (TVS) demonstrated high diagnostic accuracy for deep infiltrating endometriosis, with a sensitivity of up to 96% and specificity of 99%. EFI emerged as a valuable predictor of natural conception post-surgery. Additionally, the review underscores the frequent co-occurrence of adenomyosis in women with endometriosis, which may further compromise fertility. Despite advancements in imaging techniques and classification systems, the variability in symptom presentation and disease progression continues to challenge early diagnosis and effective management. **Conclusions:** Endometriosis is a prevalent gynecological condition affecting women of reproductive age and is associated with infertility. This paper describes the diagnostic approach to endometriosis during a first-line gynecological appointment, focusing on clinical history, physical examination, and the role of imaging, particularly ultrasound, in identifying and characterizing endometriosis lesions. The adoption of standardized classification systems such as #ENZIAN and EFI enhances disease staging and fertility prognosis, allowing for tailored treatment strategies. Despite improvements in non-invasive diagnostic methods, challenges persist in correlating symptom severity with disease extent, necessitating continued research into biomarkers and novel imaging techniques. Additionally, the frequent coexistence of adenomyosis further complicates fertility outcomes, underscoring the need for comprehensive management strategies. Further research is needed to enhance early detection strategies and optimize fertility preservation techniques for affected women.

## 1. Introduction

Endometriosis, a condition affecting 10–15% of women of reproductive age, is strongly associated with infertility [1]. This chronic inflammatory disease involves the growth of endometrial tissue (like the lining of the uterus) outside the uterine cavity, often leading to pelvic pain, dysmenorrhea, and infertility.

While the exact mechanisms remain unclear, endometriosis can disrupt fertility through various pathways, including pelvic adhesions, inflammation, and immune dysfunction [2]. In this manuscript, we will describe how to diagnose endometriosis and evaluate its severity and fertility impact during a first-line gynecological appointment. This includes a comprehensive review of patient history, clinical examination, and the role of imaging studies, particularly ultrasound, in identifying and characterizing endometriosis lesions.

Endometriosis is a chronic inflammatory disease characterized by ectopic endometrial glands and stroma outside the uterine cavity [3,4]. It primarily affects pelvic organs, including the ovaries, anterior and posterior cul-de-sac, broad and uterosacral ligaments, uterus, fallopian tubes, sigmoid colon, and appendix. Additionally, it can involve organs outside the pelvis, such as the diaphragm [1]. Approximately 10% of women of reproductive age are affected by endometriosis [5]. This condition predominantly affects young women between the ages of 25 and 45, as the growth of endometrial implants is dependent on ovarian activity [6]. Despite its high prevalence, endometriosis remains difficult to assess, with a mean time to diagnosis up to 10 years after the first symptoms [7].

Endometriosis is a multifactorial disease characterized by a highly variable clinical presentation, ranging from asymptomatic cases to severe and debilitating conditions, with symptom severity not correlating with lesion size [7,8].

The pathogenesis of endometriosis remains poorly understood. Although the retrograde menstruation theory, first proposed by Sampson in the 1920s, is widely accepted, it does not explain all clinical manifestations. Notably, while retrograde menstruation occurs in approximately 90% of women, only about 10% develop endometriosis, highlighting the involvement of additional mechanisms in disease development [3]. Another proposed mechanism is coelomic metaplasia, which involves the transformation of normal peritoneal tissue into ectopic endometrial tissue. This transformation is most likely driven by endogenous inductive stimuli which may be hormonal or immunologic [1,9]. The theory of embryonic Müllerian residues, also known as Müllerianosis, states that residual cells from the migration of the Müllerian ducts during embryonic development have the potential to differentiate into endometriotic lesions. This process is believed to be influenced by estrogen or estrogen-mimicking compounds [1,10]. A more recent theory regarding the non-uterine origin of endometriosis suggests that stem cells originating from bone marrow could differentiate into endometriotic tissue [5]. While these theories explain the ectopic location of endometrial tissue, additional mechanisms are necessary for their further development. They include escape from immune clearance, attachment to the peritoneal epithelium, invasion of the epithelium, establishment of local neurovascular networks, and continued growth and survival. The selective survival advantage of endometrial cells is facilitated by decreased apoptosis and enhanced proliferation, potentially driven by genetic mutations. Notably, the risk of severe endometriosis is six times higher in first-degree relatives of affected women compared to unaffected individuals, suggesting a hereditary component. Estrogen dependence and progesterone resistance also play critical roles. Increased activity of the aromatase enzyme and reduced expression of 17β-hydroxysteroid dehydrogenase type 2 lead to elevated bioavailability of estradiol (E2), which stimulates prostaglandin E2 production, further enhancing aromatase activity. Progesterone resistance, characterized by reduced expression of progesterone receptors, impairs the regulatory effects of progesterone [8,11]. Inflammation is a predominant mechanism of endometriosis pathogenesis and contributes to its progression. The peritoneal fluid in women with endometriosis contains increased concentrations of activated macrophages and elevated levels of pro-inflammatory cytokines and chemokines, including interleukin-6 (IL-6) [12]. These activated macrophages exhibit impaired phagocytic capacity, allowing ectopic endometrial cells to evade immune clearance and persist in the peritoneal cavity. IL-6, in particular, plays a central role by promoting a pro-inflammatory environment, enhancing the survival and proliferation of ectopic endometrial cells and contributing to angiogenesis. The persistent inflammatory milieu exacerbates tissue damage and pain, hallmarks of the disease, and perpetuates a feedback loop that supports lesion growth and survival [12,13].

Chronic pelvic pain and debilitating dysmenorrhea are the most prevalent symptoms of endometriosis. Women with endometriosis often experience deep dyspareunia, commonly described as pain in the vaginal fornix during intercourse. Dyschezia, characterized by pain immediately before or after defecation, is also a common symptom and may worsen during menstruation, occasionally leading to rectal bleeding or rectorrhagia. Endometriotic implants can also involve the bladder, causing symptoms such as dysuria and pollakiuria [3,5,14].

Accurate classification of endometriosis is crucial to assess the extent of the disease, the location of lesions, and the clinical implications of these lesions. This allows better communication between the various specialties involved in the patient’s treatment and follow-up. This can also be useful for evaluating treatment outcomes, guiding therapeutic interventions, and advancing research in this field. 

The revised American Society for Reproductive Medicine (rASRM) classification, introduced in 1996, categorizes endometriosis into four stages: minimal, mild, moderate, and severe. These stages are determined by a cumulative scoring system based on the extent of endometriotic lesions in the ovaries, peritoneum, and adhesions. While this scoring system has been globally accepted, it has notable limitations, including lack of reproducibility, discrepancies between histopathological findings and visually diagnosed stages, inconsistency between the stage of endometriosis and symptom severity, and, more importantly, its failure to account for deep infiltrating endometriosis (DIE) affecting the vagina, bowel, bladder, and uterosacral ligaments [6,15].

The #ENZIAN classification was first described in 2005 and revised in 2011 [16]. It is a useful tool for the classification of deep endometriosis in transvaginal sonography (TVS), magnetic resonance imaging (MRI), and laparoscopic staging of the disease [16,17]. The #ENZIAN classification categorizes endometriotic lesions into four compartments: vagina and recto-vaginal space (A); uterosacral ligaments (USL), cardinal ligaments, and pelvic sidewall (B); rectum (C); and far locations (F). The #ENZIAN additionally includes the involvement of the peritoneum (P), ovaries (O), and tubo-ovarian unit and the tubal patency (T) [16,17].

The compartment or organ involved in the disease is identified with capital letters, and the extent of endometriosis is classified into three stages [1,2,3].

Studies show that the extent of deep endometriosis described by the #ENZIAN classification correlates with the complexity of the surgical procedures required. This demonstrates the reliability of this scoring system in non-invasive preoperative staging of the disease. 

The Endometriosis Fertility Index (EFI) is a validated scoring system designed to stage surgically confirmed endometriosis in patients attempting non-IVF conception [18]. A significant variable in calculating the EFI score is the least function score, which is determined intraoperatively during surgical intervention. This score evaluates the functional status of the fallopian tubes, fimbriae, ovaries, and the Douglas pouch. It is calculated by summing the lowest function scores from the right and left sides. The EFI score ranges from 0 to 10, with 0 representing the poorest prognosis and 10 indicating the best prognosis for achieving pregnancy. This score serves as a predictive tool for assessing the probability of conception through non-IVF treatments following surgical staging. Notably, the EFI does not account for uterine abnormalities. The EFI is a valuable tool for developing individualized treatment plans for infertile patients with endometriosis, offering a reliable framework for clinical decision-making and prognostic evaluation [18,19,20].

## 2. Endometriosis and Infertility

Endometriosis is a well-established cause of infertility, affecting a significant proportion of patients, with infertility rates ranging from 20% to 68% [21]. The mechanisms underlying this association remain poorly understood, but several hypotheses have been proposed to explain the potential cause–effect relationship between endometriosis and infertility. Altered pelvic anatomy, often due to adhesions and fibrosis—particularly in the ovarian cortex when endometriomas are present—disrupts the normal functioning of reproductive organs, impairing egg release and tubal transport [22]. Distortion of pelvic structures can lead to tubal obstruction, reduced tubal motility, and limited access to the ovarian surface during ovulation [23]. Pelvic adhesions, commonly observed in patients with deep endometriosis (DE), further disrupt pelvic anatomy, compromising oocyte release and oocyte uptake by the fallopian tubes [24]. Additionally, chronic pelvic inflammation creates a local environment that is unfavorable for conception. Elevated levels of cytokines, growth factors, prostaglandins, and reactive oxygen species in the peritoneal fluid and lesions of patients with endometriosis interfere with ovulation, oocyte uptake, sperm function, fertilization, and embryo migration [4]. Peritoneal macrophages, which are increased in number and display a dysfunctional phenotype in women with endometriosis, contribute significantly to these processes through enhanced production of proinflammatory mediators, perpetuating pelvic inflammation and recruiting other immune cells. Moreover, endometriosis is often associated with an autoimmune component characterized by the presence of IgG and IgA antibodies and aberrant immune responses within the endometrium. These immune abnormalities may further impair implantation and embryo development [22,25].

The relationship between the severity of endometriosis and its impact on fertility is multifactorial and varies significantly among individuals. Several factors, including the extent of lesions, the presence of adhesions, the location of endometriotic tissue (such as ovarian, peritoneal, or deep infiltrating endometriosis), and the associated ovarian reserve, contribute to this variability [23,26]. Furthermore, individual molecular and immunological responses to endometriosis can influence fertility outcomes in unpredictable ways. While advanced stages of endometriosis are generally associated with more severe fertility impairment, not all patients with advanced disease experience infertility. Conversely, some patients with mild or minimal endometriosis may still face significant reproductive challenges. These findings suggest that the stage of disease alone is insufficient to predict fertility outcomes. The effects of endometriosis on fertility are not solely related to the presence of lesions but may also involve changes in the ovarian environment, fallopian tube function, and immune responses, all of which contribute to subfertility [9]. The complex interplay of these factors makes the management and prognosis of fertility in endometriosis particularly challenging. Assisted reproductive technologies (ART), including in vitro fertilization (IVF), have proven effective for many patients with endometriosis, regardless of disease severity [27,28,29]. However, success rates for ART tend to be lower in women with endometriosis compared to the general population, particularly in cases of significant ovarian or tubal involvement. A comprehensive evaluation of disease extent, ovarian reserve, and immune factors is essential for developing individualized fertility treatment plans for women affected by endometriosis [30].

## 3. Clinical Symptoms and Examination

When a patient presents with pelvic pain during a gynecological consultation, a thorough history is essential to determine the patient’s exact complaints as well as medical, surgical, obstetrical and family history, as 50% of endometriosis cases are hereditary [31,32].

The complaints described by the patient may include the following:

- dyschezia;
- dyspareunia;
- dysmenorrhea;
- menorrhagia;
- chronic pelvic pain;
- dysuria;
- other cyclical pain (such as scapulalgy or sciatalgy).

The patient is asked to rate each type of pain on a visual analog scale (VAS) [33] to assess its severity.

It has been shown that 83% of patients with endometriosis report at least one of the following symptoms: abdominopelvic pain, menorrhagia, and dyspareunia, compared with 29% of patients without endometriosis [34]. Nevertheless, the intensity of pain varies considerably from patient to patient, and the level of pain does not reflect the extent of endometriosis. Moreover, 2–50% of patients may be asymptomatic [34,35].

There is only a weak correlation between the symptoms reported by the patient and the severity of the lesions, making clinical diagnosis challenging [36,37,38]. 

After patient consent, a clinical gynecological examination is then performed, despite its limited diagnostic value.

**Figure 1:**
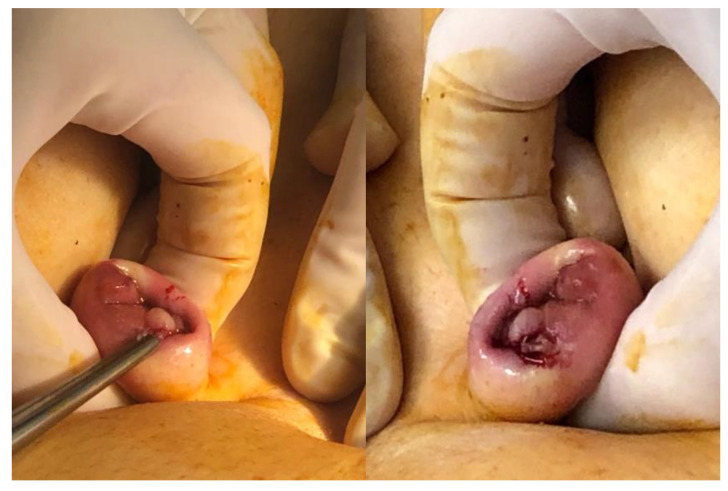
Umbilical endometriosis nodule.

Abdominal palpation can be valuable, particularly when the patient’s medical history suggests the possibility of a wall nodule, such as after a cesarean section, where palpation can help identify areas of induration. It is also crucial to examine for the presence of umbilical endometriosis (Figure 1), which may manifest as bleeding from the umbilicus during menstruation.

**Figure 2:**
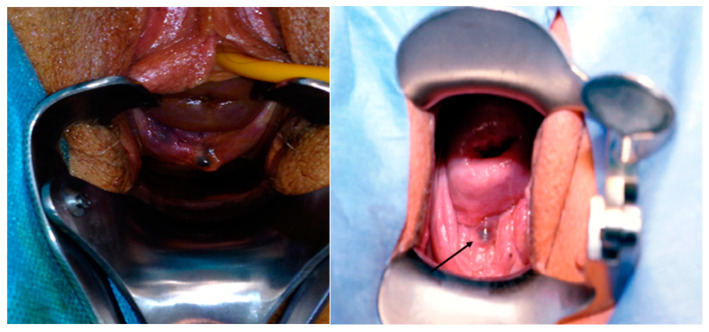
Vaginal endometriosis nodule.

Speculum examination is recommended to visualize areas of vaginal wall retraction or nodules (Figure 2) that may indicate vaginal endometriosis [39]. 

Vaginal examination aims to identify areas of retraction, induration, or with the presence of nodules [36,39]. During this examination, it is important to encourage the patient to report any pain, as this will help pinpoint the location of any lesions.

The vaginal walls, uterine position and mobility, vesico-uterine fold, utero-sacral ligaments, and recto-vaginal septum can all be evaluated. Adnexal masses may also be palpated, and their mobility may be assessed [36,39].

Additionally, a rectal examination may be conducted to check for a rectal lesion (and to estimate the distance from the anal margin, which is important for a potential resection surgery) or a lesion in the uterine parametrium [36]. 

Research indicates that even when performed by an experimented professional, the sensitivity of a clinical examination for detecting endometriosis lesions in the lower uterine segment (LUS), vagina, and rectum is only 73.5%, 50%, and 46%, respectively. This underscores the limitations of the clinical exam [40]. 

## 4. Ultrasound

During the gynecological examination, in patients presenting symptoms of pain, infertility, or clinical examination suggestive of endometriosis, a pelvic ultrasound should be performed [37,38,41]. The sensitivity of the test is enhanced by the expertise of the sonographer, as the deep posterior endometriosis sensitivity of transvaginal ultrasound is 96% and has a specificity of 99% [42,43].

**Figure 3:**
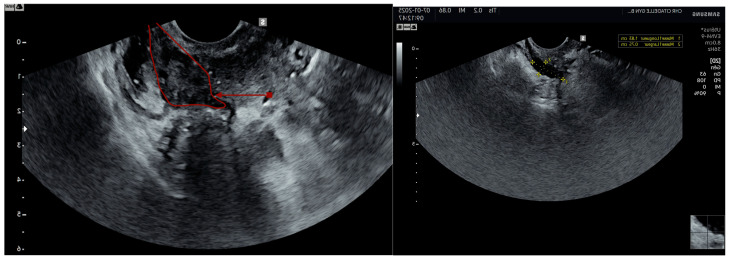
Rectovaginal isoechogenic (surrounded in red) and hypoechogenic nodule.

On ultrasound, endometriosis is characterized by hypoechoic or isoechoic (Figure 3) spots with regular or irregular borders [44].

Ultrasound should follow the International Deep Endometriosis Analysis (IDEA) group recommendations [45]: 

Criteria for assessing the uterus and adnexae:

- Uterine mobility
- Adenomyosis (MUSA score [46])
- Endometriomas (number, size, kissing ovaries)

Searching for sonographic “soft markers” and fixed ovaries:

- Look for specific tenderness in areas that increase the likelihood of adhesions and superficial endometriosis.
- Apply pressure between the ovary and uterus, the ovary and lateral pelvic wall, or between the uterosacral ligament and the ovary using a bimanual technique to identify adhesions among these structures.

Sliding signs:

- Gently press against the cervix with the transvaginal probe to check if the anterior rectum moves smoothly across the posterior cervix (retrocervical area) and the posterior vaginal wall. If the anterior rectal wall glides freely, the sliding sign is considered positive for this region.
- Assess whether the anterior bowel moves smoothly over the posterior part of the upper uterus/fundus by placing one hand on the woman’s lower anterior abdomen to ballot the uterus between the probing hand and the transvaginal probe held in the other hand. A positive finding here also confirms the sliding sign.

Deep infiltrating endometriosis nodule in anterior and posterior compartments:

- To assess the bladder, the probe should be inserted into the anterior vaginal fornix. If bladder endometriosis is suspected, advise the patient to avoid emptying her bladder before the ultrasound.
- To evaluate the posterior compartment, insert the probe into the posterior fornix.

Other ultrasound techniques may also be used when endometriosis is suspected. Abdominal ultrasonography should be conducted because ureteral lesions may not be clearly seen with endovaginal ultrasonography. This method allows assessment of the renal structure and helps rule out hydronephrosis, which often arises from ureteral damage at the parametrial level [47]. Additionally, wall nodules can be identified through abdominal ultrasound. The rectal route is infrequently utilized in practice. 

Although these imaging techniques can be followed by the ENZIAN classification, they are considered as a reliable indicator of infertility.

When endometriosis is suspected and the primary provider ultrasound is negative, referral to an endometriosis center is recommended.

Additional Examinations:

While other tests can be beneficial for identifying endometriosis lesions, detailing these supplementary examinations is beyond the scope of this practical guide for gynecological consultations. 

However, it is worth mentioning MRI, which is frequently compared to pelvic ultrasound for assessing endometriosis. MRI is typically conducted as a secondary examination when ultrasound is not very informative or is not relevant to the patient’s complaints [44]. MRI offers advantages over ultrasound, particularly in examining the lateral and posterolateral compartments, and demonstrates increased sensitivity for deep lesions or atypical endometriomas [43,48,49].

It is important to note that MRI, despite its potential benefits, has limitations in diagnosing deep endometriosis. Its accuracy is somewhat compromised by a higher false positive rate (23%) [48] and lower sensitivity (90%) and specificity (91%) compared to ultrasound [50].

Although MRI classification can be utilized, it does not serve as a predictor for infertility.

## 5. Adenomyosis

Adenomyosis is a non-cancerous condition within the uterus. It occurs when endometrial tissue grows abnormally deep into the muscular wall of the uterus. This abnormal growth often involves an overgrowth of surrounding tissue [51]. Endometriosis and adenomyosis are closely linked due to their shared underlying mechanisms. Both conditions involve the presence of endometrial tissue (glands and stroma) in unexpected locations [52]. In adenomyosis, this misplaced tissue resides within the muscular wall of the uterus (myometrium). The prevalence of adenomyosis varies significantly, ranging from 5% to a significant 70% depending on the population studied [53].

While symptoms such as pelvic pain, painful periods (dysmenorrhea), heavy bleeding (menorrhagia), and infertility can occur in 25% of adenomyosis cases [54], a surprising one-third of patients experience no symptoms at all [55].

Researchers are exploring how adenomyosis might affect fertility. Several potential mechanisms are being investigated, including disruption of the normal structure and function of the uterine muscle, altered movement (peristalsis) within the uterus that could hinder sperm transport, localized areas of increased estrogen activity, abnormal inflammatory responses, increased free radicals, and an abundance of blood vessels [56].

Given this potential link to fertility issues, it is important to consider adenomyosis during routine gynecological examinations, particularly pelvic ultrasounds. Interestingly, adenomyosis often co-exists with endometriosis, with a high rate (65–70%) of women having both conditions [57].

The identification of adenomyosis on pelvic ultrasound is based on the criteria established by the Morphological Uterus Sonographic Assessment (MUSA) group [46], which include the following features:

- ill-defined myometrial lesions, either focal or diffuse
- globular-shaped uterus
- asymmetry in the size of uterine walls
- myometrial cysts
- echogenic lesions within the myometrium
- fan-shaped shadowing patterns
- echogenic subendometrial lines and buds
- translesional vascularity
- irregular junction zone
- interrupted junctional zone (on 3D reconstruction)

These sonographic findings are crucial in the diagnosis and assessment of adenomyosis, enabling accurate evaluation of the condition’s extent and severity.

Preoperative assessments using the ENZIAN ultrasound and the ASRM surgical score do not take adenomyosis into account.

## 6. Conclusions

**Figure 4:**
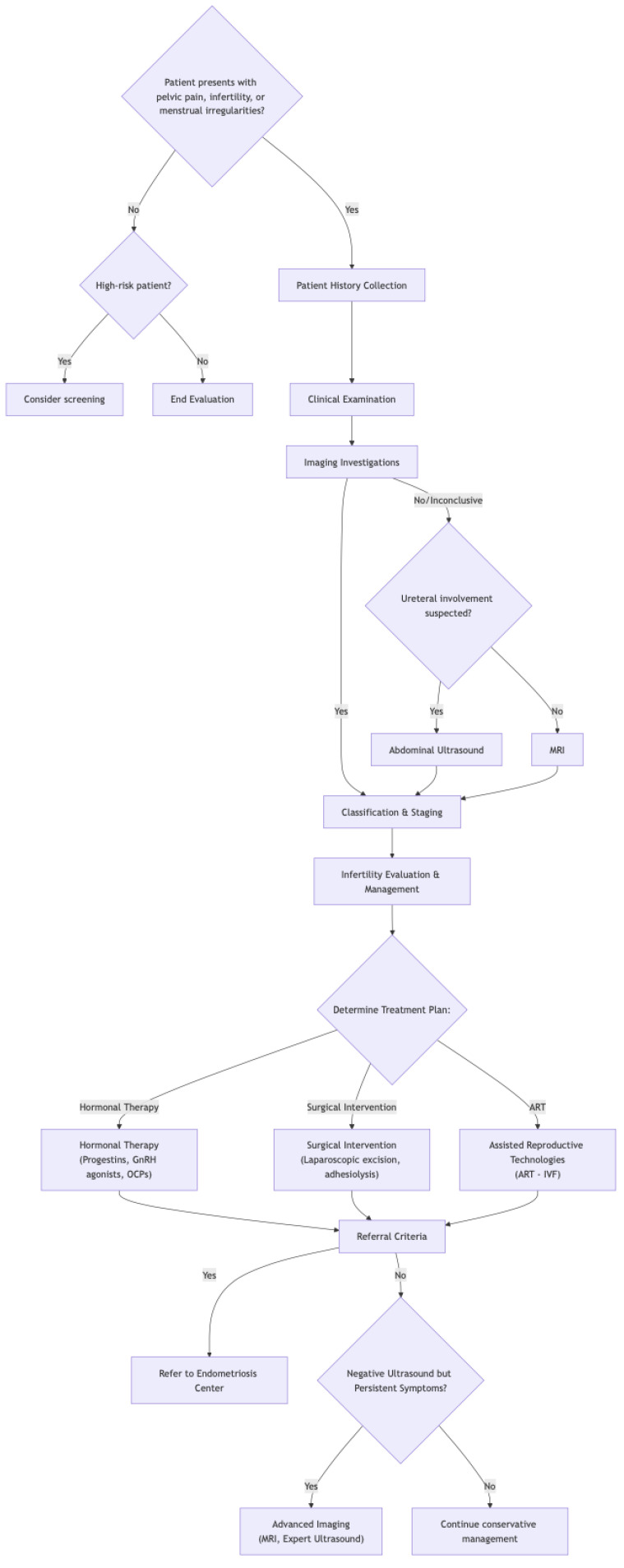
Flowchart of endometriosis diagnosis and treatment.

Endometriosis presents a significant health challenge for women, impacting fertility and quality of life. On the positive side, early diagnosis through thorough evaluation and imaging, coupled with prompt management (Figure 4), can dramatically improve outcomes. This proactive approach helps control debilitating symptoms, potentially preserves fertility, and slows disease progression. Recognizing both endometriosis and adenomyosis is vital for accurate diagnosis. However, challenges remain. Diagnosing endometriosis can be difficult, often requiring multiple steps, which can delay treatment. The complex and not fully understood nature of the disease, along with the variability in symptoms, makes effective management tricky. Plus, treatments themselves can have side effects. Despite these hurdles, the benefits of early intervention for endometriosis are clear, emphasizing the importance of continued research and improved diagnostic tools. 

## Data Availability

Not applicable.

## Abbreviations

The following abbreviations are used in this manuscript:

rASRM	The Revised American Society for Reproductive Medicine
EFI	The Endometriosis Fertility Index
TVS	Transvaginal sonography
MRI	Magnetic resonance imaging
E2	Estradiol
IL-6.	Interleukin-6
USL	Uterosacral ligaments
DE	Deep endometriosis
ART	Assisted reproductive technologies
IVF	In vitro fertilization
IDEA	International Deep Endometriosis Analysis
MUSA	Morphological Uterus Sonographic Assessment
